# Impact of the COVID-19 pandemic on internal medicine training in the United States: results from a national survey

**DOI:** 10.1186/s12913-023-10237-9

**Published:** 2023-11-22

**Authors:** Frederique St-Pierre, Romela Petrosyan, Arjun Gupta, Stephen Hughes, John Trickett, Susan Read, Vanessa Van Doren, Andrew Zeveney, Christiana Shoushtari

**Affiliations:** 1 Department of Internal Medicine, Division of Hematology/Oncology, Northwestern University, 676 N St Clair St (Suite 850), Chicago, IL 60611 USA; 2 Combined Brigham and Women’s Hospital and Massachusetts General Hospital Department of Medicine, Division of Nephrology, Harvard University, Boston, MA USA; 3 Department of Cardiology, The Mount Sinai Hospital, New York, NY USA; 4 Department of Pulmonary, Critical Care, and Sleep Medicine, Naval Medical Center San Diego, San Diego, CA USA; 5 Department of Internal Medicine, Mayo Clinic, Phoenix, AZ USA; 6 Research Center, American College of Physicians, Philadelphia, PA USA; 7 Department of Internal Medicine, Emory University School of Medicine, Atlanta, GA USA; 8 Oak Street Health, Chicago, IL USA

**Keywords:** COVID-19, Pandemic, Internal medicine, Training, Resident, Fellow

## Abstract

**Background:**

Internal medicine (IM) residency is a notoriously challenging time generally characterized by long work hours and adjustment to new roles and responsibilities. The COVID-19 pandemic has led to multiple emergent adjustments in training schedules to accommodate increasing needs in patient care. The physician training period, in itself, has been consistently shown to be associated with vulnerability with respect to mental well-being. The impact of the COVID-19 pandemic on the experience of IM trainees is not well established.

**Objective:**

Characterize the impact of the COVID-19 pandemic on trainee clinical education, finances, and well-being.

**Methods:**

We developed a survey composed of 25 multiple choice questions, 6 of which had an optional short-answer component. The survey was distributed by the American College of Physicians (ACP) to 23,289 IM residents and subspecialty fellows. We received 1,128 complete surveys and an additional 269 partially completed surveys.

**Results:**

The majority of respondents reported a disruption in their clinical schedule (76%) and a decrease in both didactic conferences (71%) and protected time for education (56%). A majority of respondents (81%) reported an impact on their well-being with an increase in their level of burnout and 41% of respondents reported a decrease in level of direct supervision. Despite these changes, the majority of trainee respondents (78%) felt well prepared for clinical practice after graduation.

**Conclusions:**

These results outline the vulnerable position of internal medicine physicians in training. Preserving educational experiences, adequate supervision, and humane work hours are essential in protecting trainees from mental illness and burnout during global emergencies.

**Supplementary Information:**

The online version contains supplementary material available at 10.1186/s12913-023-10237-9.

## Introduction

Internal medicine (IM) residency is well-recognized as a challenging period of time generally characterized by long work hours, adjustment to new roles and responsibilities, and a steep clinical learning curve [1]. Multiple studies have established that depression and burnout are highly prevalent among IM trainees and are even higher during the intern year, the first year of post-graduate training [1–3]. One study estimated the burnout rate among internal medicine residents to be as high as 63% based on responses to the Maslach Burnout Inventory [4]. Other studies have found that up to 49% of residents screen positively for major depression with by PHQ-9 criteria [1, 2]. Suicide is the second most common cause of death among residents in programs accredited by the Accreditation Council of Graduate Medical Education (ACGME), second only to all forms of cancer combined [5]. Residency also represents a financially vulnerable time for many trainees due to an average medical school debt burden of $200,000 [6], average interest rate of 6.5% for federal loans [7], and an average PGY-1 salary of $58,921 in 2020 [8]. The COVID-19 pandemic has presented unforeseen challenges to institutions, physicians, and other members of the healthcare team [9]. With respect to trainees, this pandemic has led to multiple emergent adjustments in training schedules to accommodate increasing needs in patient care and has resulted in major changes in educational curricula to meet Center of Disease Control (CDC) guidelines for group interactions [10, 11]. The impact of the COVID-19 pandemic on the experience of IM trainees is not well defined. The objective of this study is to characterize the impact of the COVID-19 pandemic on clinical education, finances and well-being among IM residents and IM subspecialty fellows.

## Methods

This is a cross-sectional, survey-based study. The survey/questionnaire was developed by the representatives of the American College of Physicians (ACP) IM trainee council and by experienced staff members of the ACP, the largest IM specialty society in the U.S. The survey items were reviewed for content and structure by methodologic experts at the ACP and at the home institutions of the authors. The survey was composed of 7 optional demographic questions and 25 multiple-choice questions related to the trainee experience during the pandemic, 6 of which had an optional short-answer component to further characterize details of the trainee experience. The questionnaire was divided into the following sections: Training Environment and Education, Well-being, and Financial Impact. The full questionnaire is available in Supplemental Appendix 1. Survey items were uploaded into an online survey platform (Snap Surveys) for web-based distribution. The survey was distributed by the ACP to its active members training in IM at an ACGME-accredited program at the time of survey distribution, including IM residents and IM subspecialty fellows. The one-time use link was distributed through members’ individual email addresses, preventing multiple survey entries by single participants. The survey was distributed from March 12 to May 26, 2021 to 23,289 IM trainees. Survey respondents were entered into a random drawing for one of 10 electronic gift cards of 50 dollars, which were funded by a Clinician Scholars Program at a large academic center. Non-respondents were sent reminders for survey completion at four, two, and one weeks remaining until survey closing. In all, 1,128 complete responses were received, with an additional 269 partial responses. Only data from fully completed surveys (n = 1,128) was included in the final analysis. All responses remained anonymous and no directly identifiable information was collected. The study was deemed exempt by the academic center’s institutional review board (IRB #21–000109).

Initial statistical analysis was performed through Excel using the Likert scale for quantitative, multiple-choice questions and demographic questions. Qualitative, short-answer responses were collected and grouped into recurring themes for analysis and discussion. The short-answer and demographic questions were considered optional, and failure to respond to these questions did not constitute an incomplete survey. Surveys were considered fully completed if all non-optional questions were answered. Categorical and continuous data were further compared using the Kruskal–Wallis test and a One-way ANOVA with Bonferroni correction. STATA version SE 18.0 (College Station, TX) was used for all analyses. Two-tailed *P* values < 0.05 were considered statistically significant.

## Results

Respondent Characteristics.

Respondents varied by post-graduate year (PGY) training level, with the majority of respondents in PGY-2 and 3 (resident) years (*n* = 734, 65%), followed by PGY-1 (intern) year (*n* = 285, 25%), and PGY-4 to 8 (fellow) years (*n* = 109, 10%). The majority of fellow respondents were in their PGY-4 year (*n* = 63, 58%). Geographic distribution of respondents reflected the distribution of internal medicine residency programs nationally. There was an approximately even distribution between male and female respondents, with a small percentage of participants identifying as non-binary or other genders. Approximately 40% of respondents were international medical graduates (IMGs). Respondent characteristics are outlined in Table 1. Overall response rate to the survey was 5% (1,128/23,289).

**Table 1:** Demographics of internal medicine resident and fellow participants by training level

**Characteristic**	**PGY-1** *N* = 285 (%)	**PGY-2 & 3** *N* = 734 (%)	**PGY 4–8** *N* = 109 (%)
Post-graduate year			
PGY-1	285 (100)	0	0
PGY-2	0	403 (55)	0
PGY-3	0	331 (45)	0
PGY-4	0	0	63 (58)
PGY-5	0	0	24 (22)
PGY-6	0	0	16 (15)
PGY-7	0	0	5 (5)
PGY-8	0	0	1 (1)
Region			
West	42 (15)	98 (13)	20 (18)
Midwest	79 (28)	169 (23)	27 (25)
Northeast	84 (29)	232 (32)	24 (22)
South	80 (28)	235 (32)	38 (35)
Age group (y)			
19 – 24	3 (1)	0	0
25 – 30	218 (76)	421 (57)	27 (25)
31 – 35	51 (18)	250 (34)	59 (54)
36 – 40	10 (4)	46 (6)	17 (16)
41 +	3 (1)	17 (2)	6 (6)
Race/Ethnicity			
Non-Hispanic White	100 (35)	292 (40)	54 (50)
African/African American	15 (5)	43 (6)	4 (4)
Hispanic	24 (8)	57 (8)	7 (6)
Asian/Asian American	52 (18)	111 (15)	18 (17)
Indian	39 (14)	82 (11)	18 (17)
Arabic	14 (5)	34 (5)	3 (3)
Pakistani	14 (5)	48 (7)	3 (3)
Pacific Islander	1 (0)	3 (0)	0
Native American/Alaskan Native	1 (0)	0	0
Other	11 (4)	27 (4)	5 (5)
Prefer not to disclose	26 (9)	75 (10)	6 (6)
Gender			
Male	135 (47)	348 (47)	53 (49)
Female	134 (47)	345 (47)	51 (47)
Non-Binary	1 (0)	2 (0)	1 (1)
Other	0	1 (0)	0
Prefer not to disclose	15 (5)	38 (5)	4 (4)
Undergraduate Medical Education			
US / Canada	161 (57)	420 (57)	62 (57)
IMG	112 (39)	288 (39)	45 (41)
Prefer not to disclose	12 (4)	26 (4)	2 (2)

### Training environment and education

The majority of trainees (*n* = 852, 76%) reported a disruption in their clinical rotation schedule, a third of whom reported a disruption in their clinical environment lasting more than 6 months. Commonly reported changes included decrease in teaching time on clinical rounds (*n* = 745, 66%), fewer didactic conferences (*n* = 804, 71%), and less protected time for education (*n* = 636, 56%). 89% (*n* = 1,007) of trainee respondents worked in a COVID unit during the pandemic. Those who reported a decrease in protected time for education (*n* = 636) were asked what their programs had done to address COVID-19 related reductions to their clinical education. 63% (*n* = 398) of respondents indicated that program leadership held meetings to discuss the changes in education, 62% (*n* = 393) reported that new resources were shared or created by their program leadership and chief residents (mainly characterized as virtual initiatives in the short-answer component of the question), and 9% (*n* = 57) of trainees wrote in the short-answer component that to their knowledge, nothing had been done to remediate the changes in rotation and educational schedule.

**Fig. 1:**
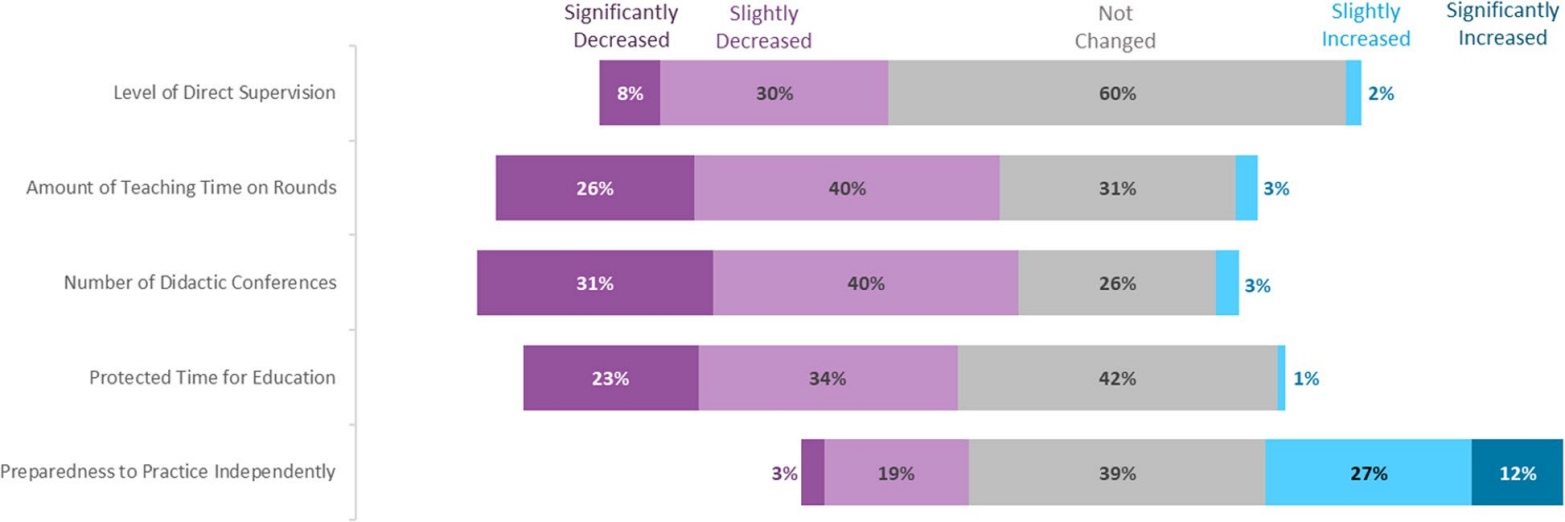
Effects of the COVID-19 pandemic on training environment and education

**Fig. 2:**
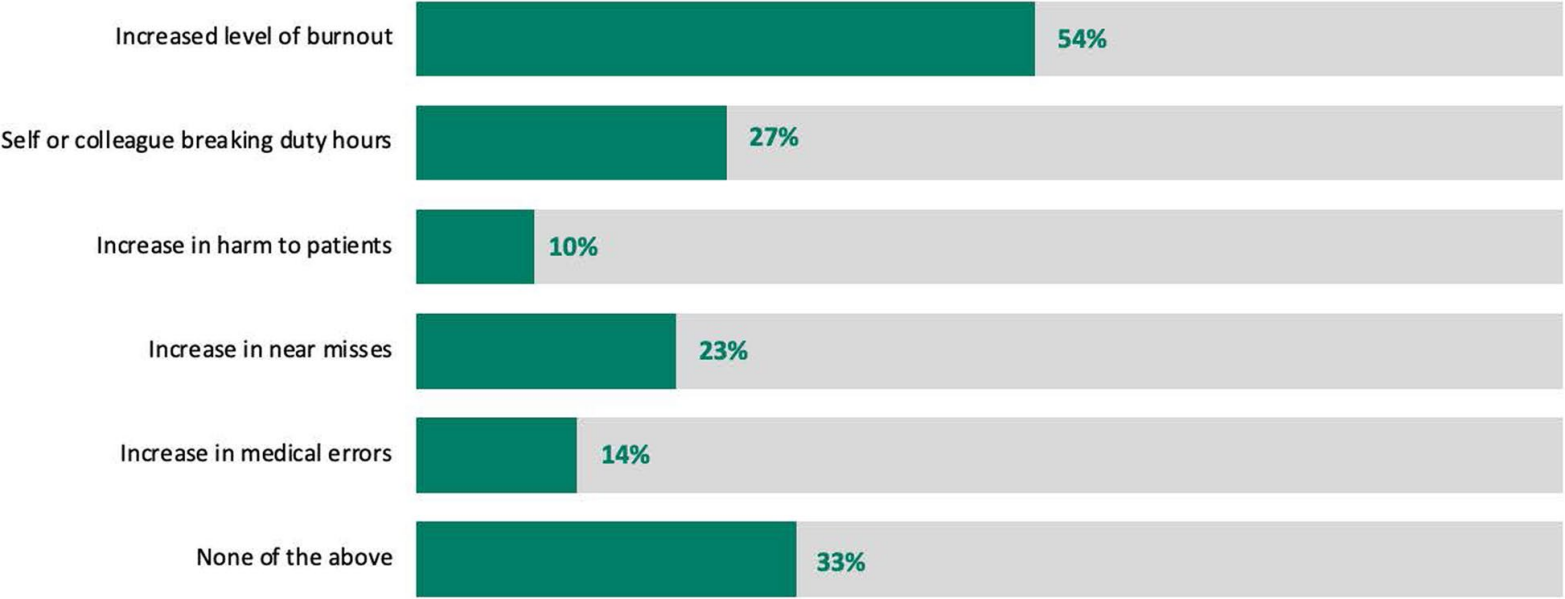
Perceived consequences of decreased supervision

The majority of respondents did not report changes to direct supervision (*n* = 672, 60%), while 38% (*n* = 428) reported a decrease in direct supervision. Among those reporting a decrease in direct supervision (*n* = 428), 82% (*n* = 353) felt that there was insufficient increase in indirect supervision to account for the decrease in direct supervision, and 28% (*n* = 119) reported increase in medical errors, near misses, and/or patient harm as a result. 54% (*n* = 231) of trainees who experienced a decrease in supervision reported that this resulted in increased level of burnout, and 27% (*n* = 116) reported that this led to themselves or colleagues breaking duty hours. These results are illustrated in Figs. 1 and 2.

Despite the disruption in clinical training reported by many trainees, the majority of trainee respondents felt that they were as prepared or more prepared for independent practice after graduation (*n* = 883, 78%). 50% (*n* = 560) of respondents reported that their career choice was not influenced by the pandemic, while 20% (*n* = 222) of respondents reported being more likely to choose a subspecialty.

### Well-being

Overall, 81% (*n* = 909) of respondents felt the pandemic has increased their level of burnout. Utilizing the Kruskal–Wallis Test, the level of burnout differed among trainees depending on their level of seniority (*p* = 0.005). When comparing interns (PGY-1) versus residents (PGY-2 and 3), residents had a greater level of burnout which was statistically significant (41.6% vs 31.2% respectively, *p* = 0.001). Fellows (PGY-4 and beyond) also had a lower rate of reported burnout compared to residents, although this difference did not quite reach statistical significance (33% vs 41.6% respectively, *p* = 0.066). These results are outlined in Table 2. Significantly increased burnout appeared to occur more frequently in the Northeast (45%) and West (40%) regions compared to Midwest (32.4%) and South (35.1%) regions (*p* = 0.0064). Results are highlighted in Table 3. IMGs did not report higher burnout rates compared to US/Canadian trainees (Supplemental Table 2). Rate of significant burnout was higher in female trainees compared to their male counterparts, and the highest rate of significant burnout was among the small number of trainees (*n* = 4) who identify as non-binary (44.1% vs 31.7% vs 50%, *p* = 0.0009) (Supplemental Table 3). Hispanic trainees reported higher rates of significant burnout compared to other races/ethnicities (59% vs 36.3% for Caucasian, 37.5% for African American, 31.9% for Asian/Pacific Islander, and 38.7% for Middle-Eastern (*p* = 0.0013) (Supplemental Table 4).

**Table 2:** Level of burnout by trainee level

Level of Burnout	Intern *n* = 285 (%)	Resident (PGY-2 & 3) *n* = 734 (%)	Fellow (PGY-4 and senior) *n* = 109 (%)	*p*-value
Significantly Less	0	4 (0.5)	1 (0.9)	0.3692
Slightly Less	6 (2.1)	7 (9.5)	7 (6.4)	0.0002
Neutral	62 (21.8)	117 (15.9)	15 (13.8)	0.0530
Slightly More	128 (44.9)	301 (41)	50 (45.9)	0.3964
Significantly More	89 (31.2)	305 (41.55)	36 (33)	0.0049

**Table 3:** Level of burnout by region

	Midwest *n* = 275 (%)	Northeast *n* = 340 (%)	South *n* = 353 (%)	West *n* = 160 (%)	*p*-value
Significantly Less	0	3 (0.9)	2 (0.6)	0	0.3158
Slightly Less	4 (1.45)	5 (1.5)	9 (2.5)	2 (1.2)	0.6132
Neutral	55 (20)	45 (13.2)	72 (20.4)	22 (13.8)	0.0274
Slightly More	127 (46.2)	134 (39.4)	146 (41.4)	72 (45)	0.3281
Significantly More	89 (32.4)	153 (45)	124 (35.1)	64 (40)	0.0064

**Fig. 3:**
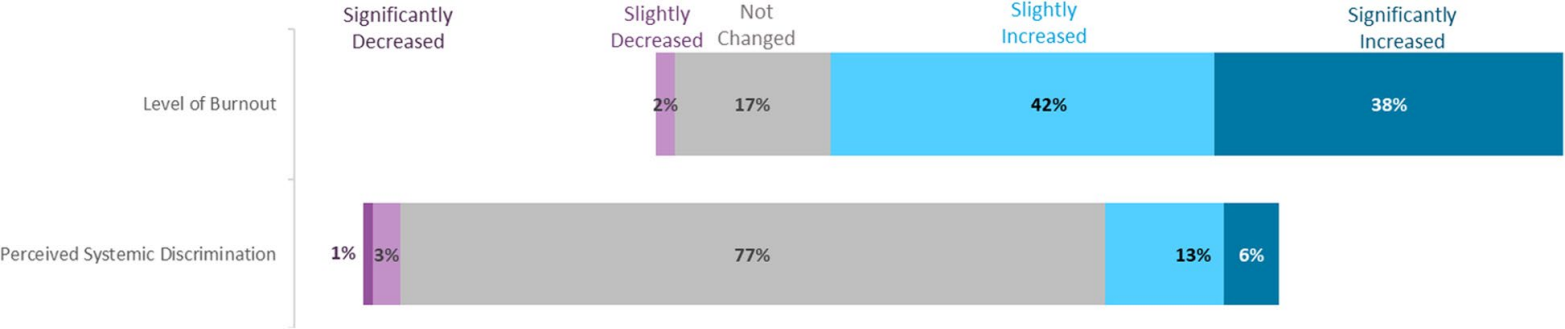
Effects of the COVID-19 pandemic on burnout and perceived systemic discrimination

**Fig. 4:**
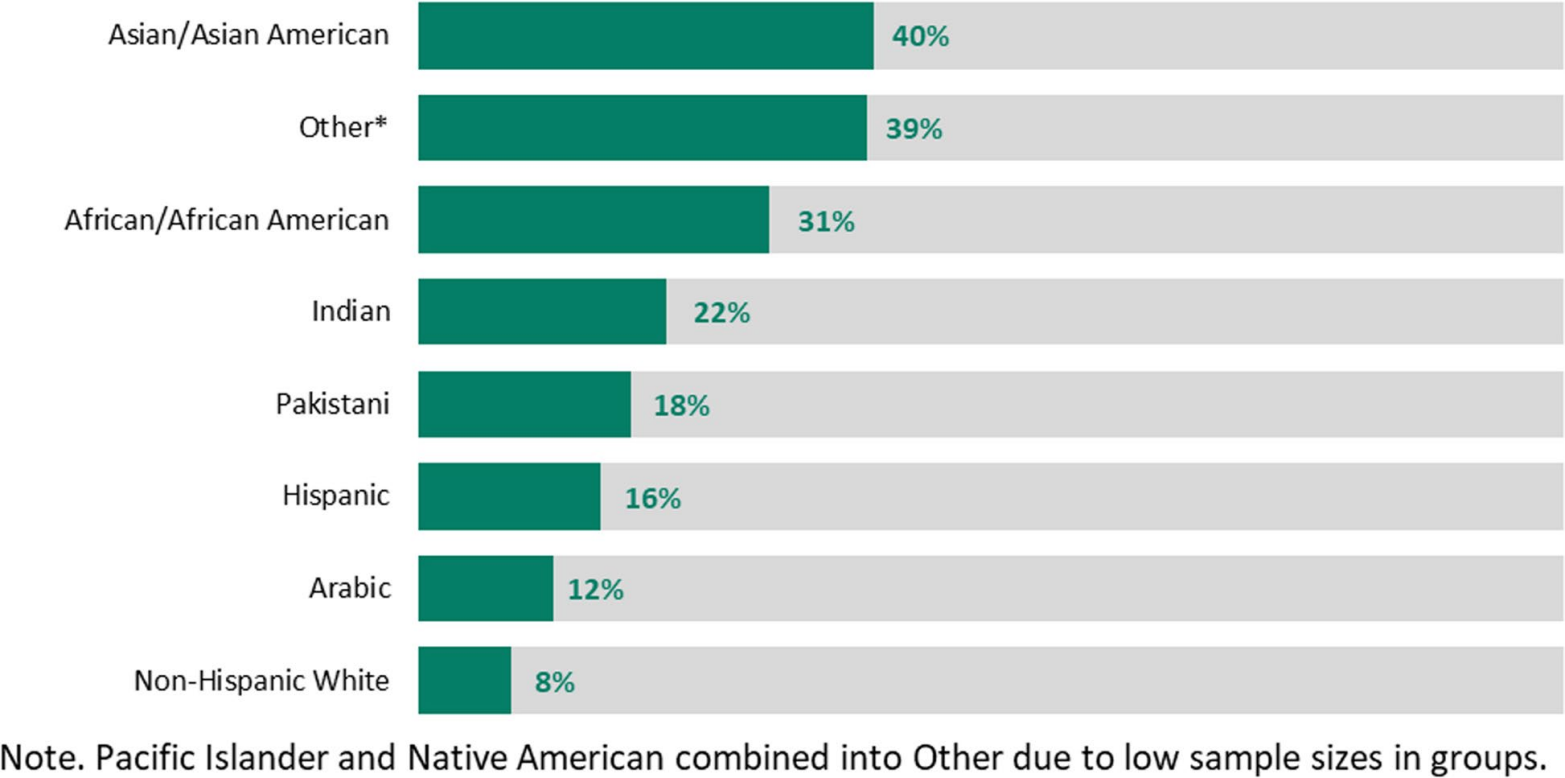
Perceived systemic discrimination by race/ethnicity

19% (*n* = 217) of trainee respondents reported an increase in experience of discrimination, possibly including but not restricted to discrimination based on gender, race/ethnicity, or age. Groups with highest perceived discrimination were Asian/Asian American and African/African American trainees, with 40% (72/181) and 31% (19/62) of trainees in these groups, respectively, reporting a perceived increase in discrimination. Figures 3 and 4 illustrate these findings.

Nearly all trainee respondents have known someone personally who was diagnosed (*n* = 1,065, 94%), hospitalized (*n* = 606, 54%), or died (*n* = 325, 29%) due to COVID-19. Many trainees have had an immediate family member diagnosed (*n* = 235, 21%), be hospitalized (*n* = 72, 6%), or die (*n* = 27, 2%) due to COVID-19. 11% (*n* = 128) of trainees had an extended family member pass away due to COVID-19.

### Financial Impact

68% (*n* = 763) of trainee respondents reported no additional reimbursement or pay as compensation for the changes in schedule, education, and work hours incurred during the pandemic. 4% (*n* = 45) of residents reported additional reimbursement offered to residents and fellows who were considered high risk, and 19% (*n* = 213) reported additional reimbursement was offered to all residents and fellows equally, regardless of exposure risk. 6% (*n* = 64) of respondents reported additional pay for extra months worked on COVID-19 units. 54% (*n* = 612) of trainee respondents indicated being aware of additional reimbursement being offered to non-physician healthcare professionals at their institution to compensate for additional work and hardship associated with the pandemic. 37% (*n* = 422) of trainees responded that staff physicians at their institution received additional reimbursement for their work during the COVID-19 pandemic, and 15% (*n* = 174) of trainees reported that this occurred irrespective of individual risks or exposures. Among the institutions that provided additional compensation to staff physicians (*n* = 422), 57% (*n* = 241) also offered additional compensation to residents and fellows. With respect to the perceived financial impact on respondents’ institutions, 37% (*n* = 420) of trainee respondents reported a negative financial impact, 14% (*n* = 158) reported no significant financial impact, and 49% (*n* = 550) of trainees reported not knowing how the pandemic affected their institution financially.

## Discussion

These survey results outline the high prevalence of alterations to the IM trainee training experience and curriculum during the COVID-19 pandemic. Almost universally, respondents to our survey have reported a disruption in their clinical rotation schedule associated with a reduction in teaching time on rounds and a decrease in academic lectures. The most commonly reported accommodation by programs was a transition to virtual conferences and remote education, but most trainees still reported that the overall protected time for education was affected by the pandemic. An important cause for these changes is likely the sudden increase in volume of hospitalized patients during the pandemic, taking residents and fellows away from some of their typical outpatient or elective rotations in order to support these critically ill patients. A decrease in available and appropriate personnel due to COVID exposures and illnesses also likely contributed to the emergent need for reallocation of house staff. Finally, with the CDC guidelines recommending avoidance of large group gatherings, the conventional in-person didactic lectures were abruptly forced to adapt to alternate methods of teaching. Over 30% of IM trainees reported these changes lasted for 6 months or more, and almost 10% of trainees reported minimal effort by their program to augment training with alternate educational sessions.

In conjunction with this, a high proportion of trainees reported a decrease in the level of direct supervision by attending physicians, with generally insufficient increases in compensatory indirect supervision and support. We hypothesize that the decrease in supervision likely occurred secondary to the surge of critically ill patients and increased physician absence from work due to COVID-related exposure or illness. There was a clear indication from respondents that a perceived increase in medical errors and decrease in quality of patient care ensued from this change in direct supervision. It is encouraging that 60% of IM trainees did not experience a change in direct supervision, highlighting that deficits may be institutionally dependent and that effective leadership may ensure proper supervision despite these extraordinary circumstances. It is also encouraging that despite the overall loss in protected time for education, most trainees are still confident in their clinical acumen and feel ready to enter independent practice. That being said, we cannot exclude the possibility that trainee self-assessment may be impaired by the lack of direct observation and supervision in the setting of the pandemic. Our survey did not assess the respondents’ confidence in entering independent outpatient/primary care practice in comparison to hospital medicine, however, we suspect that in programs where outpatient rotations were discontinued in favor of hospital rotations to meet the high demands of these healthcare systems, this distinction may be important. This is supported by the relatively high proportion of residents who reported being more likely to choose a subspecialty as a result of the pandemic.

Importantly, this survey outlines the extremely high burnout rates among residents and fellows during the pandemic. The increase in work hours, rapid and unpredictable change in clinical schedule, and added stress from a decrease in direct supervision are all likely contributing factors. In addition to the personal burden associated with caring for critically ill patients experiencing COVID-19, the direct burden that COVID-19 has had on trainees and their families has been substantial. Aside from the direct impact of illness, the pandemic has forced family separations between trainees and their extended families, bearing in mind social distancing in addition to institutional and national travel restrictions. PGY-2 and 3 residents reported higher rates of burnout compared to PGY-1 s. We hypothesize that in addition to already having many accumulated months of high work hours prior to the pandemic, more experienced residents may have quickly been given a higher level of responsibility without proportionate supervision or support because of the rapidly evolving healthcare needs during the pandemic. Additionally, the last two years of residency are often used to hone in on a specialty of choice and focus on learning opportunities that are directly relevant to a trainee’s career goal. The COVID pandemic may have impaired this to a greater level in residents who are only one or two years away from subspecialty training or from being an attending.

Despite the substantial changes in schedules and work hours, an overwhelming majority of residents and fellows received no additional pay or compensation. This may be explained by the overall financial strain the COVID-19 pandemic incurred on most healthcare systems, although our survey results indicated that many institutions provided hazard pay to their staff physicians while a similar benefit was not awarded to trainees. This is consistent with results from a recent study which surveyed IM program directors, showing that only 19.5% of US IM programs provided hazard pay for their trainees during the pandemic [12]. Our survey also reported a perceived higher proportion of additional compensation to non-physician healthcare workers in comparison to residents and fellows. Failure of institutions to compensate their trainees for increased work hours while other healthcare workers at the same institution are being compensated for their efforts could result in additional psychological distress and frustration.

Of note, this survey demonstrated that 19% of respondents reported an increase in their experience of systemic discrimination. This disproportionately affected Asian/Asian American (40%; 72/181) and African/African American (31%; 19/62) trainees. Racial microaggressions during the pandemic have been documented in other studies, especially discrimination experienced by Asian healthcare workers and Asian communities, possibly in part due to the virus originating from Wuhan, China, but probably with much higher underlying complexity [13, 14].

The low response rate (5%) to the survey represents an important limitation to this study. ACP’s recorded survey response rate among residents and fellows has varied between 15 and 31% over the years, with their most recent survey receiving a response rate of 21% in 2018. The 10–15% absolute reduction in usual response rate may be explained by the increased workload and ongoing challenges faced by the target population. An additional limitation is the distribution of the survey to ACP members, which may have excluded residents from smaller community programs that do not offer complimentary ACP membership to their trainees. Furthermore, although this survey was validated by experts, it could not be piloted due to time constraints in the setting of the global pandemic. Finally, assessment of burnout was done through self-reporting, and standardized instruments such as the Maslach Burnout Inventory [15] were not employed due to constraints in resources and time.

Despite a low response rate, we believe these results to be generalizable to the U.S. IM training system, bearing in mind that most of the highlighted issues are highly institution-dependent. Our respondent characteristics show a relatively even geographic distribution of respondents. The gender distribution and distribution of IMG respondents is also proportionate to reported IM resident demographics by American Board of Internal Medicine (ABIM) (43% female and 41% IMG in 2019–2020) [16, 17]. The distribution of race/ethnicity among respondents corresponds approximately to the IM trainee demographics reported by the Association of American Medical Colleges (AAMC) (35.5% Non-Hispanic White, 24% Asian, 6.7% Hispanic, 4.7% Black) [17]. These results cannot be interpreted outside of the U.S. residency and fellowship system, although there is evidence from several single-institutional and regional studies in various countries suggesting similar issues worldwide, including decreased protected educational time and decreased psychosocial wellbeing among medical residents [11, 18, 19]. Future research should focus on quality improvement initiatives aiming to protect IM trainees from mental illness and burnout for the remainder of this pandemic and throughout the evolution of Graduate Medical Education. This could include evaluating strategies to ensure the preservation of education and supervision for trainees under any circumstance, and to promote a culture focused on resident wellness, mutual respect, and humane work hours. Further survey-based research should also be conducted to elicit the causes of systemic discrimination in the healthcare setting during challenging societal events.

## Conclusion

This national survey among IM trainees in the U.S. highlights important issues that affected residents and fellows during the COVID-19 pandemic. Specific areas of concern include decreased protected educational time, decreased direct supervision at some institutions, an alarmingly high level of burnout among trainees, and increased systemic racism during the pandemic. Finding ways to preserve education and supervision under any circumstance, and promoting a culture focused on resident wellness, mutual respect, and humane work hours, will be essential in protecting trainees from mental illness and burnout for the remainder of this pandemic and throughout the evolution of Graduate Medical Education in the future.

### Supplementary Information

**Additional file 1: Supplemental Appendix 1.** Survey.

**Additional file 2: Supplemental Table 1.** Level of Burnout by Post-Graduate Year.  **Supplemental Table 2.** Level of Burnout by Undergraduate Medical Education.  **Supplemental Table 3.** Level of Burnout by Gender. **Supplemental Table 4.** Level of Burnout by Race/Ethnicity.

**Additional file 3. **

## Data Availability

The dataset(s) supporting the conclusions of this article is(are) included within the article (and its additional file(s).

## Abbreviations

ACP: American college of physicians
IM: Internal medicine
IMG: International medical graduate
PGY: Post-graduate year
