# Supplementary material for: Impact of the COVID-19 pandemic on internal medicine training in the United States: results from a national survey
Source: BMC Health Serv Res. 2023 Nov 22;23:1285. doi: 10.1186/s12913-023-10237-9 (PMC10666403; doi:10.1186/s12913-023-10237-9)
Supplement: Supplementary file 1 — Additional file 1: Supplemental Appendix 1. Survey. [file 12913_2023_10237_MOESM1_ESM.docx]

Supplemental Appendix 1 – Survey

Question 1: **Since the start of the COVID-19 pandemic, my clinical rotation schedule has been disrupted for:** *(i.e. change in days off, change in clinical schedule, working on COVID units, etc.*)**:**

- 1. Has not been disrupted
  2. 1 month
  3. 2 months
  4. 3 months
  5. 4 months
  6. 5 months
  7. 6 months
  8. 7 months
  9. 8 months
  10. 9 months
  11. 10 months
  12. 11 months
  13. 1 year
  14. More than one year
  15. Since the beginning of the pandemic

Question 2: **During the COVID-19 pandemic I have been asked to rotate on a unit for patients with COVID-19 for:**

- 1. Not rotated on patient for COVID-19 patients
  2. 1 month
  3. 2 months
  4. 3 months
  5. 4 months
  6. 5 months
  7. 6 months
  8. 7 months
  9. 8 months
  10. 9 months
  11. 10 months
  12. 11 months
  13. 1 year
  14. More than one year
  15. The entire pandemic

Question 3: **During the COVID-19 pandemic I have been asked to serve in an attending role or serve as the internal medicine ‘consult’ for a COVID unit staffed by other specialties for:**

- 1. I haven’t served in these roles
  2. 1 month
  3. 2 months
  4. 3 months
  5. 4 months
  6. 5 months
  7. 6 months
  8. 7 months
  9. 8 months
  10. 9 months
  11. 10 months
  12. 11 months
  13. 1 year
  14. More than one year
  15. The entire pandemic

Question 4: **Since the start of COVID-19, my level of direct supervision (i.e. either at rounds or in-person check-ins after rounds) by attending physicians has:**

- 1. Significantly decreased
  2. Slightly decreased
  3. Not changed
  4. Slightly increased
  5. Significantly increased

Question 5: ***If responded A or B in Q4:* Has your level of indirect supervision (i.e. calls to run lists, check-in, etc.) increased to account for decrease in synchronous supervision?**

- 1. It has increased enough to account for decrease in direct supervision
  2. It has increased but doesn’t make up for the loss of direct supervision
  3. It has not increased or decreased
  4. It has moderately decreased
  5. It has significantly decreased

Question 6: ***If responded A or B in Q4:* This reduction of supervision by attending physicians since the start of COVID-19 has:** *Choose all that apply*

- 1. Increased medical errors
  2. Led to near misses
  3. Led to harm for patients
  4. Led to myself or colleagues breaking duty hours
  5. Increased my level of burnout
  6. None of the above

Question 7: **Since the start of the COVID-19 pandemic, how, if at all, have these changes in clinical training (setting and supervision) affected your clinical development as an internist?**

- 1. I feel significantly more prepared to practice independently after graduation
  2. I feel somewhat more prepared to practice independently after graduation
  3. I feel neither more prepared or less prepared to practice independently after graduation
  4. I feel somewhat less prepared to practice independently after graduation
  5. I feel significantly less prepared to practice independently after graduation

Question 8: **Since the start of the COVID-19 pandemic, how, if at all, have these changes in clinical training (setting and supervision) affected your likely career choices?** *Choose all that apply*

- 1. Schedule changes during COVID led me to miss out on training in a future specialty I was considering
  2. I am less likely to choose any subspecialty training
  3. I am less likely to remain in clinical medicine
  4. I am more likely to choose a subspecialty, other than hospital medicine or pulmonology
  5. I am more likely to choose hospital medicine
  6. I am more likely to choose pulmonology for subspecialty training
  7. My career choices have not changed

Question 9: **How has the COVID-19 pandemic changed your clinical education, in the amount of teaching time on rounds?**

- 1. Significantly less teaching time on clinical rounds
  2. Slightly less teaching time on clinical rounds
  3. No change in teaching time on clinical rounds
  4. Slightly more teaching time on clinical rounds
  5. Significantly more teaching time on clinical rounds

Question 10: **How has the COVID-19 pandemic changed your clinical education, in the number of didactic conferences (scheduled lectures from attendings**)**?**

- 1. Significantly fewer didactic conferences
  2. Slightly fewer didactic conferences
  3. No change in didactic conference frequency
  4. Slightly more didactic conferences
  5. Significantly more didactic conferences

Question 11: **How has the COVID-19 pandemic changed your clinical education, in the amount of protected time for education?**

- 1. Significantly less protected time for education
  2. Slightly less protected time for education
  3. No change in protected time for education
  4. Slightly more protected time for education
  5. Significantly more protected time for education

Question 12: ***If answered A, B, D or E:* What has your program done to address COVID-19 related reductions to your clinical education?** *Choose all that apply*

- 1. Our program leadership (PD, APDs) have held meetings to discuss changes in our education
  2. Our program leadership and/or chiefs have shared resources for our education
  3. Our chief residents have created new resources to improve our education
  4. Other
     - *Please specify*

Question 13: **Have you or those who support you been diagnosed with COVID-19?** *Choose all that apply*

- 1. I have been diagnosed with COVID-19
  2. One or more of my co-residents have been diagnosed with COVID-19
     1. If yes, please quantify
  3. One or more of my co-workers (not co-resident/fellows) have been diagnosed with COVID-19
  4. One or more of my program leaders (chiefs, APDs, PDs) have been diagnosed with COVID-19
  5. One or more members of my immediate family have been diagnosed with COVID-19
  6. One or more members of my extended family have been diagnosed with COVID-19
  7. One or more members of my community (neighborhood, religious group, etc…) have been diagnosed with COVID-19
  8. No one I know personally has been diagnosed with COVID-19

Question 14: ***If answered anything other than #8 in Q13:*** **Have you or those who support you been hospitalized with COVID-19?** *Choose all that apply*

1. I have been hospitalized due to COVID-19
2. One or more of my co-residents have been hospitalized due to COVID-19
   - - If yes, please quantify
3. One or more of my co-workers (not co-residents/fellows) have been hospitalized due to COVID-19
4. One or more of my program leaders have been hospitalized due to COVID-19
5. One or more members of my immediate family have been hospitalized due to COVID-19
6. One or more members of my extended family have been hospitalized due to COVID-19
7. On or more members of my community (neighborhood, religious group, etc…) have been hospitalized due to COVID-19
8. No one I know personally has been hospitalized with COVID-19

Question 15: ***If answered anything other than #8 in Q14:*** **Have any of those who support you died of COVID-19 related illness?** *Choose all that apply*

1. One or more of my co-residents have died, attributable to COVID-19
   - If yes, please quantify
2. One or more of my co-workers (not co-residents/fellows) have died, attributable to COVID-19
3. One or more of my program leaders died, attributable to COVID-19
4. One or more members of my immediate family died, attributable to COVID-19
5. One or more members of my extended family died, attributable to COVID-19
6. One or more members of my community died, attributable to COVID-19
7. No one I know personally has died of COVID-19 related illness

Question 16: **How has the COVID-19 pandemic changed your level of burnout?**

1. Significantly increased my level of burnout
2. Slightly increased my level of burnout
3. Has not changed my level of burnout
4. Slightly decreased my level of burnout
5. Significantly decreased my level of burnout

Question 17: **How do you believe the COVID-19 pandemic has changed the level of systemic discrimination you experience** (such as for your gender, race/ethnicity, age, or other characteristics)**?**

1. Significantly increased my experience of discrimination
2. Slightly increased my experience of discrimination
3. Has not changed my experience of discrimination
4. Slightly decreased my experience of discrimination
5. Significantly decreased my experience of discrimination

Question 18: **Has your hospital offered additional reimbursement as a result of the COVID-19 pandemic to Residents and Fellows?**

- 1. Not to any residents and fellows
  2. Residents and fellows equally in the program, regardless of risk or exposures
  3. Residents and fellows who were considered high risk
  4. Residents and fellows based on the months they worked on COVID-19 units
  5. Some residents and fellows but by other criteria
     - Please specify

Question 19: **Has your hospital offered additional reimbursement as a result of the COVID-19 pandemic to *Staff Physicians (i.e. fully licensed attending physicians working on teaching or non-teaching services)?***

- 1. Not to any staff physicians
  2. Staff physicians, regardless of risk or exposures
  3. Staff physicians who were considered high risk
  4. Staff physicians based on the months they worked on COVID-19 units
  5. Some staff physicians but by other criteria
     - Please specify

Question 20: **Has your hospital offered additional reimbursement as a result of the COVID-19 pandemic to non-physician clinical staff (i.e. health professionals working with patients other than physicians)?**

- 1. Not to any clinical staff other than physicians
  2. All clinical staff other than physicians equally
  3. Clinical staff other than physicians who were considered high risk
  4. Clinical staff other than physicians on the months they worked on COVID-19 units
  5. Some clinical staff other than physicians but by other criteria
     - Please specify

Question 21: **How, if at all, has your hospital been impacted by financial issues as a result of the COVID-19 pandemic?** *Choose all that apply*

- 1. No financial impacts of which I am aware
  2. Uncertain
  3. Is likely to close as a result of COVID-19 related reimbursement losses
  4. May not be able to hire as many graduates of my program due to COVID-19
  5. Cannot hire graduates of my program due to COVID-19
  6. Cannot make upgrades or expansion due to COVID-19
  7. Other financial impacts
  8. Closed as a result of COVID-19 related reimbursement losses

Question 22: **What is the COVID-19 test turnaround time at your institution, today?**

- 1. Within 4hrs
  2. Within 12hrs
  3. Within 24hrs
  4. 1 day
  5. 2 days
  6. 3 days
  7. 4 days
  8. 5 days
  9. 6 days
  10. 7 days
  11. 8 days
  12. 9 days
  13. 10 days
  14. More than 10 days

Question 23: **What has been the longest expected COVID-19 testing result time at your clinic during the pandemic?**

- 1. Within 4hrs
  2. Within 12hrs
  3. Within 24hrs
  4. 1-2 days
  5. 1-3 days
  6. 2-4 days
  7. 3-5 days
  8. 5-7 days
  9. 7-9 days
  10. 10 or more days

Question 24: **What have been the effects of COVID-19 test scarcity (due to both testing availability and testing turnaround) at your institution?** C*hoose all that apply*

- 1. Delays in diagnosis of patients with COVID
  2. Delays in diagnosis of patients with other conditions
  3. Challenging conversations between patient and clinician
  4. Reduction in patients’ trust in health care system
  5. Delays in treatment for patients with COVID
  6. Delays in treatment for patients with other conditions
  7. Increased levels of medical errors
  8. Increased levels of burnout among clinicians and staff
  9. My institution did not experience problems with test availability or turnaround times
  10. Other
      - *Please specify*

Question 25: **In general, do you consider limitations in COVID-19 testing for clinical staff (yourself and others) to be a failure of your clinical site to protect the healthcare workforce?**

- 1. Yes
     - *If yes, please explain*
  2. No
