# Supplementary material for: Impact of the COVID-19 pandemic on internal medicine training in the United States: results from a national survey
Source: BMC Health Serv Res. 2023 Nov 22;23:1285. doi: 10.1186/s12913-023-10237-9 (PMC10666403; doi:10.1186/s12913-023-10237-9)
Supplement: Supplementary file 2 — Additional file 2: Supplemental Table 1. Level of Burnout by Post-Graduate Year. Supplemental Table 2. Level of Burnout by Undergraduate Medical Education. Supplemental Table 3. Level of Burnout by Gender. Supplemental Table 4. Level of Burnout by Race/Ethnicity. [file 12913_2023_10237_MOESM2_ESM.docx]

Supplemental Table 1. Level of Burnout by Post-Graduate Year

| Level of Burnout | PGY-1  n=285 (%) | PGY-2  n=403 (%) | PGY-3  n=331 (%) | PGY-4  n=63 (%) | PGY-5  n=24 (%) | PGY-6  n=16 (%) | PGY-7  n=5 (%) | PGY-8  n=1 (%) | p-value |
| --- | --- | --- | --- | --- | --- | --- | --- | --- | --- |
| Significantly Less | 0 | 2 (0.5) | 2 (0.60) | 1 (1.6) | 0 | 0 | 0 | 0 | 0.8299 |
| Slightly Less | 6 (2.1) | 5 (1.24) | 2 (0.60) | 2 (3.2) | 1 (4.2) | 0 | 3 (60) | 1 (100) | 0.0000 |
| Neutral | 62 (21.8) | 68 (16.9) | 49 (14.8) | 7 (11.1) | 3 (12.5) | 4 (25) | 1 (20) | 0 | 0.2956 |
| Slightly More | 128 (44.9) | 170 (42.2) | 131 (39.6) | 29 (46) | 11 (45.8) | 9 (56.2) | 1 (20) | 0 | 0.6255 |
| Significantly More | 89  (31.2) | 158 (39.2) | 147 (44.4) | 24 (38.1) | 9 (37.5) | 3 (18.8) | 0 | 0 | 0.0129 |

Supplemental Table 2. Level of Burnout by Undergraduate Medical Education

| Level of Burnout | US/Canada  n=643 (%) | IMG  n=445 (%) | Did not Disclose  n=40 (%) | p-value |
| --- | --- | --- | --- | --- |
| Significantly Less | 2 (0.31) | 1 (0.22) | 2 (5) | 0.0001 |
| Slightly Less | 11 (1.71) | 8 (1.8) | 1 (2.5) | 0.9338 |
| Neutral | 95 (14.8) | 92 (20.7) | 7 (17.5) | 0.0401 |
| Slightly More | 292 (45.4) | 175 (39.3) | 12 (30) | 0.0364 |
| Significantly More | 243 (37.8) | 169 (38) | 18 (45) | 0.6590 |

Supplemental Table 3. Level of Burnout by Gender

| Level of Burnout | Female  n=530 (%) | Male  n=536 (%) | Non-binary  n=4 (%) | Other  n=1 (%) | p-value |
| --- | --- | --- | --- | --- | --- |
| Significantly Less | 1 (0.2) | 1 (0.2) | 0 | 0 | 0.0000 |
| Slightly Less | 9 (1.7) | 10 (1.9) | 1 (25) | 0 | 0.0090 |
| Neutral | 57 (10.8) | 126 (23.5) | 0 | 0 | 0.0000 |
| Slightly More | 229 (43.2) | 229 (42.7) | 1 (25) | 1 (100) | 0.4160 |
| Significantly More | 234 (44.1) | 170 (31.7) | 2 (50) | 0 | 0.0009 |

Supplemental Table 4. Level of Burnout by Race/Ethnicity

| Level of Burnout | Caucasian n=446 (%) | African American n=56 (%) | Hispanic n=78 (%) | Asian/Pacific Islander n=163 (%) | Middle Eastern n=385 (%) | p-value |
| --- | --- | --- | --- | --- | --- | --- |
| Significantly Less | 1 (0.2) | 0 | 0 | 1 (0.6) | 3 (0.8) | 0.7050 |
| Slightly Less | 7 (1.6) | 2 (3.6) | 0 | 4 (2.5) | 7 (1.8) | 0.5602 |
| Neutral | 62 (13.9) | 12 (21.4) | 12 (15.4) | 38 (23.3) | 70 (18.2) | 0.0654 |
| Slightly More | 214 (48) | 21 (37.5) | 20 (25.6) | 68 (41.7) | 156 (40.5) | 0.0032 |
| Significantly More | 162 (36.3) | 21 (37.5) | 46 (59) | 52 (31.9) | 149 (38.7) | 0.0013 |
